# Association between Cannabinoid CB_1_ Receptor Expression and Akt Signalling in Prostate Cancer

**DOI:** 10.1371/journal.pone.0065798

**Published:** 2013-06-05

**Authors:** Mariateresa Cipriano, Jenny Häggström, Peter Hammarsten, Christopher J. Fowler

**Affiliations:** 1 Pharmacology, Department of Pharmacology and Clinical Neuroscience, Umeå University, Umeå, Sweden; 2 Umeå School of Business and Economics, Department of Statistics, Umeå University, Umeå, Sweden; 3 Pathology, Department of Medical Biosciences, Umeå University, Umeå, Sweden; Nathan Kline Institute for Psychiatric Research and New York School of Medicine, United States of America

## Abstract

**Background:**

In prostate cancer, tumour expression of cannabinoid CB_1_ receptors is associated with a poor prognosis. One explanation for this association comes from experiments with transfected astrocytoma cells, where a high CB receptor expression recruits the Akt signalling survival pathway. In the present study, we have investigated the association between CB_1_ receptor expression and the Akt pathway in a well-characterised prostate cancer tissue microarray.

**Methodology/Principal Findings:**

Phosphorylated Akt immunoreactivity (pAkt-IR) scores were available in the database. CB_1_ receptor immunoreactivity (CB_1_IR) was rescored from previously published data using the same scale as pAkt-IR. There was a highly significant correlation between CB_1_IR and pAkt-IR. Further, cases with high expression levels of both biomarkers were much more likely to have a more severe form of the disease at diagnosis than those with low expression levels. The two biomarkers had additive effects, rather than an interaction, upon disease-specific survival.

**Conclusions/Significance:**

The present study provides data that is consistent with the hypothesis that at a high CB_1_ receptor expression, the Akt signalling pathway becomes operative.

## Introduction

The endocannabinoid (eCB) system, comprising the G-protein coupled CB_1_ and CB_2_ receptors, their endogenous ligands anandamide and 2-arachidonoylglycerol, and their synthetic and catabolic enzymes, has been shown to be involved in the control of proliferation, migration and invasive behaviour of a wide variety of cancer cells [1]–[5]. The human prostate expresses functionally active CB_1_ receptors [6], and anandamide reduces the rate of epidermal growth factor- (EGF) and prolactin-stimulated growth of human prostate cancer cell lines in a manner involving activation of CB_1_ receptors [7], [8]. In contrast, both mitogenic and, at higher concentrations, antiproliferative effects of cannabinoids have been seen for unstimulated prostate cancer cells [9]–[14], as well as effects not related to interactions with CB receptors [2], [14]–[16]. A variety of mechanisms, including production of ceramide, down-regulation of EGF receptors (EGFR) and prolactin receptors, inhibition of RhoA activity and sustained activation of extracellular signal-regulated kinases (Erk) have been implicated in the inhibitory effects of cannabinoids upon prostate cancer cell growth or motility [7], [8], [12], [17].

Manipulation of the levels of 2-arachidonoylglycerol and related homologues (by blockade or knockdown of the catabolic enzyme monoacylglycerol lipase, which is responsible for the hydrolysis of these lipids) reduces survival, mobility and the invasive properties *in vitro* as well as growth *in vivo* of androgen-insensitive PC-3 prostate cancer cells in a manner mediated in part by CB_1_ receptors [18], [19]. Conversely, inhibition of the synthesis of 2-arachidonoylglycerol or transfection of cells with fatty acid amide hydrolase (FAAH), the enzyme responsible for the hydrolysis of anandamide, increases the invasivity of PC-3 cells in vitro [18], [20]. These data are all consistent with the notion that the eCB system, in addition to having a wealth of other regulatory properties in the body [21], plays a role in the local control of cancer cell spread.

The converse of the regulatory role of the eCB system described above is the possibility that the eCB system is dysfunctional in tumours and that this contributes to disease pathogenesis. Several studies have reported that markers of the eCB system show a changed expression in cancer types such as mantle cell lymphoma and colorectal cancer [22]–[24], although the direction of the change is not always the same. In a small cohort of cases with hepatocellular cancer, for example, a low expression of CB_1_ receptors is associated with a poorer outcome than in cases with a high expression of CB_1_ receptors [25], whereas the reverse is true for pancreatic cancer [26] and stage II microsatellite-stable colorectal cancer [24]. In the case of prostate cancer, both CB_1_ receptors and FAAH are overexpressed in the tumour tissue compared with non-malignant luminal epithelial tissue or cells [20], [27]–[29] and in a large well-characterised cohort of patients with a long follow-up, we noted a higher frequency of severe cases (Gleason score 8–10) among those cases with a high tumour CB_1_ receptor and/or FAAH immunoreactivity [27], [29]. Further, for cases followed by expectancy after diagnosis, tumour CB_1_ receptor immunoreactivity (CB_1_IR) provides robust prognostic information with respect to disease-specific survival that is additive to that provided by the Gleason score [27]. Thus, for example, for cases with Gleason scores 6–7 at diagnosis, the 15-year probabilities of event-free survival for CB1IR scores < *vs.* ≥ the median score, respectively, were 85±9% vs. 44±9% [27].

Whilst the study above clearly associates a high CB_1_ receptor expression with disease severity and outcome in prostate cancer, it provides no mechanistic information as to why this is the case. One possible mechanism is that the eCB system “switches” from being damaging to prostate cancer cells at low to moderate CB_1_ receptor expression levels to promoting their survival at high expression levels. The theoretical basis for this suggestion comes from a study using mouse astrocytoma cells [30]. In this study, cells were transfected with plasmids containing a reporter gene (eGFP, a green fluorescent protein) and either none, CB_1_ or CB_2_ receptors. Clones with low to moderate or high receptor levels were then selected and treated with the CB receptor agonist CP55,940. At low to moderate receptor levels, the agonist caused apoptosis due in part to a prolonged stimulation of the Erk signalling pathway. However, the clones with high receptor expression levels showed additionally an activation of the Akt signalling pathway in response to the CB receptor agonist. This pathway is a “survival” pathway regulating cell proliferation and apoptosis, and the cells with high CB receptor expression levels did not apoptose in the presence of the CB receptor agonist, unless Akt was concomitantly inhibited [30].

Although the above study provides an elegant explanation as to why a high CB receptor expression can be detrimental, it is based upon selected transfected astrocytoma clones rather than prostate cancer. However, the report that the cannabinoid receptor agonists Δ^9^-tetrahydrocannabinol and *R*-(+)-methananandamide increase the production of phosphorylated Akt (pAkt) in PC-3 cells in a manner blocked by the CB_1_ receptor inverse agonist rimonabant, and that the concentration of Δ^9^-tetrahydrocannabinol used (100 nM) increases PC-3 cell proliferation [10] indicates an “Akt-switch” (i.e. a coupling of CB_1_ receptors to the Akt signalling pathway) is operative in this cell line under the conditions used by those authors.

If the association of a high CB_1_ receptor expression with prostate cancer disease severity and outcome is the result of a switch to Akt-mediated signalling, certain predictions can be made: Firstly, cases with a high tumour CB_1_ receptor expression would be expected to have higher activity in the Akt pathway and therefore there should be a positive correlation between CB_1_IR scores and pAkt-IR scores in the tumour tissue. Secondly, tumour proliferation rates should be higher in cases with high CB_1_IR and pAkt-IR (i.e. where a “switch” may be operative) than in cases where either one or both of these two variables is low. In consequence, in the present study, we have used data from our prostate cancer tissue microarray to explore the interaction between CB_1_ receptors and the Akt signalling pathway.

## Methods

### Patient material and immunochemistry

The tumour pEGFR-IR and pAkt scores used in the present study were taken from our database and have been published previously [31], [32]. The tissue material (formalin-fixed, paraffin-embedded samples) was collected at the Regional Hospital, Västerås, Sweden, between 1975 and 1991 from a total of 419 patients diagnosed with prostate cancer at transurethral resection for lower urinary tract symptoms [33]. The patients were followed until 2003. Tissue microarrays were constructed and in general between 1 and 8 cores (usually 5, including both primary and secondary Gleason grade areas; tumour tissue) and 1–4 cores (non- malignant tissue) could be scored for the parameter in question. The cores were scored on the basis of intensity and distribution to give a composite value ranging between 0–4 for pAkt-IR and 0–5 for pEGFR-IR 0–5 [31], [32]. Hitherto unpublished data for tumour ErbB2-IR (range 0–4) was also available in the database. In all cases, the cores were scored by investigators who were blind to the patient data. Clinical data (the Gleason score, the local tumour score, the presence of metastases at diagnosis as assessed by a bone scan, and the percentage of the specimen that contained tumour (%ca)) and the Ki67 index, a measure of cell proliferation were all available in the database (see [33], [34] for published reports on some of this material). The research ethical committee at Umeå university hospital (Regional Ethical Review Board in Umeå, Sweden) approved of the study and waived the need for informed consent.

Given that the original CB_1_IR scores were returned by a single investigator using a 0–3 scale [27], we elected to rescore the immunostained tumour cores (“nCB_1_IR”) but this time using digitised images, two independent investigators (MC, CJF) and the same intensity range as used for pAkt-IR [32]. The distributions at each intensity were scored as 0, 25, 50, 75 or 100%. Thus, for example, a core scored at 25% intensity 1, 25% intensity 2 and 50% intensity 3 for the biomarker in question would score 0.25×1+0.25×+0.5×3 = 2.25. One investigator (MC) also scored the non-malignant cores. For the 2594 cores scored by both investigators (including a few non-malignant cores inadvertently scored by CJF), an intra-class correlation analysis using a mixed model and testing for consistency gave a Chronbach's alpha of 0.94, and 1837 (71%) of the cores were scored ≤0.5 units of each other. Given that cores with staining somewhere between the intensity units (0, 1, 2, 3 and 4) and the distribution tranches (0, 25, 50, 75 and 100%) used are not uncommon, a difference in scores of up to 1 can be accepted. A total of 95 (3.7%) of the cores had differences in scores greater than 1. The differences in these cases could be due, for example, due to typographical errors or patterns of immunoreactivity that were difficult to score. In consequence these were rescored, again independently and without accessing the previous scores. Following the rescoring, 6 cores were discarded due to poor quality (i.e. not deemed score-able by both investigators), 88 cores now had scores within 1 unit of each other, and only 1 core with a score difference greater than 1 remained. This core was therefore excluded. The large number of cores analysed reflected the fact that several slides were stained more than once, since technical error (lack of staining) was suspected in the initial three to four rows of the first set of cores on each slide. This was confirmed when the individual scores were analysed: the distribution of scores for positions 1–4 for the first set of cores was considerably left-shifted (i.e. many scores of 0–1 returned) in the first run compared to the subsequent runs, whereas the distributions of the scores for positions 5-end (either 7 or 8, depending upon the slide) were very similar for the first and subsequent runs. In consequence, the scores for all the positions for the first run and first set of scores were discarded. Median values were then determined for the scores for each investigator (only cores being scored by both investigators were considered), and the scores were then averaged before being added to the database. The correlation between the original CB_1_IR tumour scores (scale 0–3) and the nCB_1_IR tumour scores (scale 0–4) was very high (Spearman's rho = 0.81, P<0.0001, n = 364), and the optimal (Youden) score identified by a Reporter Operated Characteristic (ROC) curve of the nCB_1_IR data for patients treated with expectancy after diagnosis with a 15 year cut-off was at a split at scores of ≤2.75 and >2.75.

### Statistics

Three statistical software programmes were used. The intraclass correlation coefficients, Cox proportional-hazards regression and two-step cluster analyses were conducted using IBM SPSS software versions 20 and 21. The ordinal regression analyses were undertaken using software developed within the R project for statistical computing (version 2.15.2) [35]. All other statistical calculations were undertaken using the statistical package built into the GraphPad Prism 5 and 6 computer programmes for the Macintosh (GraphPad Software Inc., San Diego, CA, USA). For survival analyses, an event was defined as death due to prostate cancer and entered into the database as “event = 1”, thereby allowing the determination of disease-specific survival. Death from other causes was censored, as were cases where the patient was alive at the date of last follow-up. Three cases where the disease outcome was unknown were excluded from the survival analyses.

## Results

### Correlation of CB_1_IR and pAkt-IR in the prostate cancer tissue microarray samples

In Table 1, Spearman's rho values are given for the correlation between nCB_1_IR and pAkt-IR scores in the tumour samples. If a high tumour CB_1_ receptor expression results in a switch to an Akt pathway [30], a positive correlation between the nCB_1_IR and pAkt-IR would be expected. This was indeed found (Spearman's rho = 0.29, P<0.0001, n = 274) for the tumours. A very similar value was seen (Spearman's rho = 0.27) when the original CB_1_IR scores [27] were used in place of nCB_1_IR. In theory, this correlation, although significant, could be induced between two independent variables by a third factor: if parameter A, for example, induced both pAkt (by utilising it as a downstream signalling molecule) and at the same time increased the synthesis of CB_1_ receptors, a correlation between pAkt-IR and nCB_1_IR would be seen. An obvious candidate in this respect would be EGF receptors and the closely related ErbB2 receptors, given that they couple to Akt signalling in prostate cancer cells [36], [37]. Indeed, in the tissue microarray, the pEGFR-IR and ErbB2-IR scores were correlated with pAkt-IR in the tumour tissue (Table 1 and [32]). However, the first- and second-order correlation coefficients for pAkt-IR and CB_1_IR, calculated as described in [38], remained significant when controlled for pEGFR-IR and ErbB2-IR (Table 1). There was no significant correlation between the non-malignant pAkt-IR and nCB_1_IR scores (Spearman's rho = 0.066, n = 232, P = 0.32).

**Table 1:** Zero-, first- and second order Spearman's rho values for correlations between nCB_1_IR, pEGFR-IR or ErbB2-IR and pAkt-IR.

pAkt-IR vs:	Control for:	Spearman's ρ	n	P value
nCB_1_IR	none	0.29	274	<0.0001
	pEGFR-IR	0.28	222	<0.0001
	ErbB2-IR	0.21	263	0.0005
	pEGFR-IR, ErbB2-IR	0.22	218	0.0014
pEGFR-IR	none	0.27	227	<0.0001
	nCB_1_IR	0.20	222	0.0028
	ErbB2-IR	0.22	223	0.0011
	nCB_1_IR, ErbB2-IR	0.18	218	0.0095
ErbB2-IR	none	0.36	271	<0.0001
	nCB_1_IR	0.29	263	<0.0001
	pEGFR-IR	0.37	223	<0.0001
	nCB_1_IR, pEGFR-IR	0.30	218	<0.0001

First- and second-order correlation coefficients were determined as described in [38].

**Figure 1:**
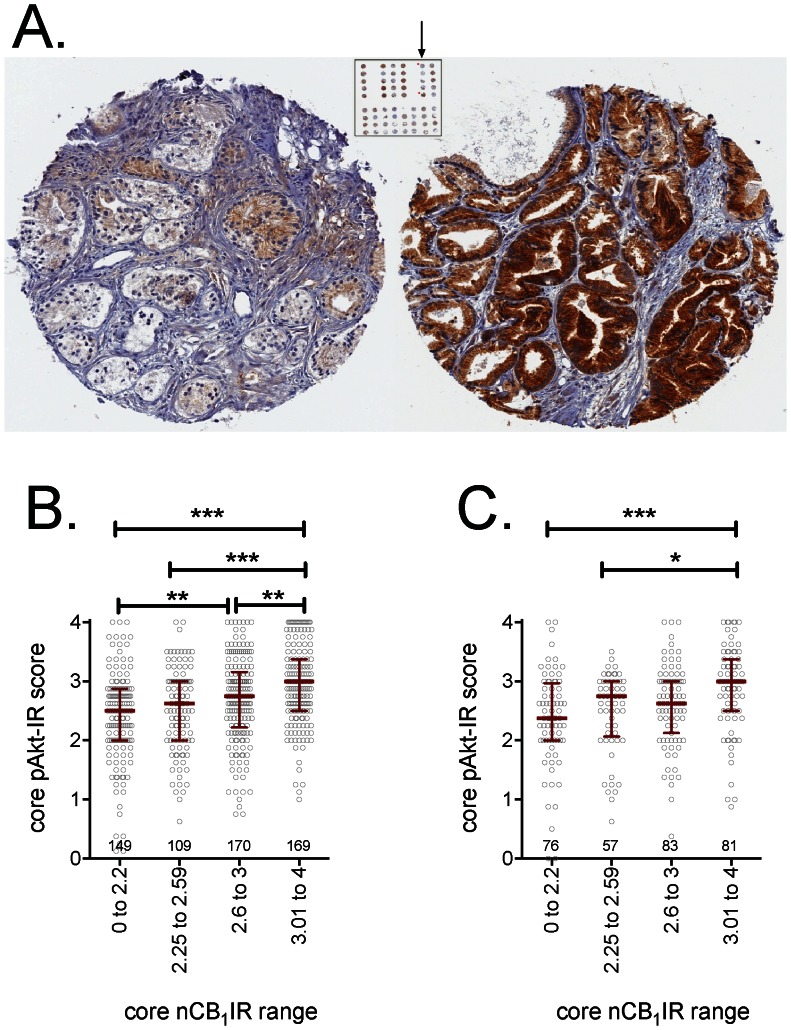
Tumour nCB_1_IR: variation with pAkt-IR. Panel A shows two tumour cores from the same case (Gleason score 7) showing a large variation in the CB_1_IR intensity. They had positions 1 and 5 of the tumour series arrowed. The left core was scored 0.75 (50% score 0, 25% score 1, 25% score 2) by one investigator, and 1 (25% score 0, 50% score 1, 25% score 2) by the other. The right core was scored 2.75 by both investigators (50% score 2, 25% score 3, 25% score 4 by one investigator; 25% score 2, 75% score 3 by the other investigator). Panel B and C show the pAkt scores for individual cores in the test (n = 595, Panel B) and validation (n = 297, Panel C) datasets, divided into approximate nCB_1_IR quadrants. The number of cases in each quadrant is shown in the graph, together with the median and interquartile ranges. *P<0.05, **P<0.01, ***P<0.001 for the comparisons shown, otherwise not significant (Dunn's multiple comparison test following significant (P<0.0001) Kruskal-Wallis test. The spearman's rho for correlations between the core nCB_1_IR and the core pAkt-IR were 0.29 and 0.28 (both P<0.0001) for the test and validation sets, respectively.

The individual scores used in this correlation were obtained from 1–8 tumour cores per case (see Methods). However, given that the cores comprising a tumour case consist of both primary and secondary Gleason grade areas [33], it is possible that a correlation might be seen at the level of the core, i.e. that a core from a given case with a low CB_1_IR will have a lower pAkt-IR than another core from the same case with a high CB_1_IR (see Fig. 1A for examples of two cores from the same case with dramatically different CB_1_IR). In consequence, we investigated the correlation between nCB_1_IR and pAkt-IR in the individual cores. In view of the large number of cores scored for both parameters (n = 892), we could divide the data randomly into a test set (n = 594) and validation set (n = 298) of cores. In both sets, a significant correlation was seen, with cores in the top quartile of CB_1_IR having a significantly greater pAkt-IR than those in the bottom two quartiles (Fig. 1B,C).

Association studies do not indicate causality, but information on this can be gleaned from the data by use of non-parametric regression techniques. A sample (80%) of the dataset was chosen at random and a non-linear multiple regression (npreg in the R statistical package) was then run to see which variables were relevant and which were not. This process was repeated 1000 times, to identify robustly associated variables. When pAkt-IR was the dependent variable in response to nCB_1_IR and pEGFR-IR, the two independent variables were never excluded. The same was true when pAkt-IR was the dependent variable in response to nCB_1_IR and ErbB2-IR, and when nCB_1_IR was the dependent variable and pAkt-IR and either pEGFR-IR or ErbB2-IR were the independent variables. These data do not add information on causality. However, when nCB_1_IR was the dependent variable and pAkt-IR, pEGFR-IR and ErbB2-IR were independent variables, pAkt-IR was excluded in 100% of the cases (the other two variables were never excluded). In the reverse analysis (when pAkt-IR was the dependent variable and nCB_1_IR, pEGFR-IR and ErbB2-IR the independent variables), nCB_1_IR was excluded in 80% of the cases, while the other two variables were never excluded. Since the exclusion rate was higher for pAkt-IR with nCB_1_IR as the dependent variable than for nCB_1_IR with pAkt-IR as the dependent variable, the data suggest that the scenario whereby activation of Akt is downstream of CB_1_ receptors is more likely than the reverse direction.

### Association of tumour nCB_1_IR and pAkt-IR with the disease severity at diagnosis

**Figure 2:**
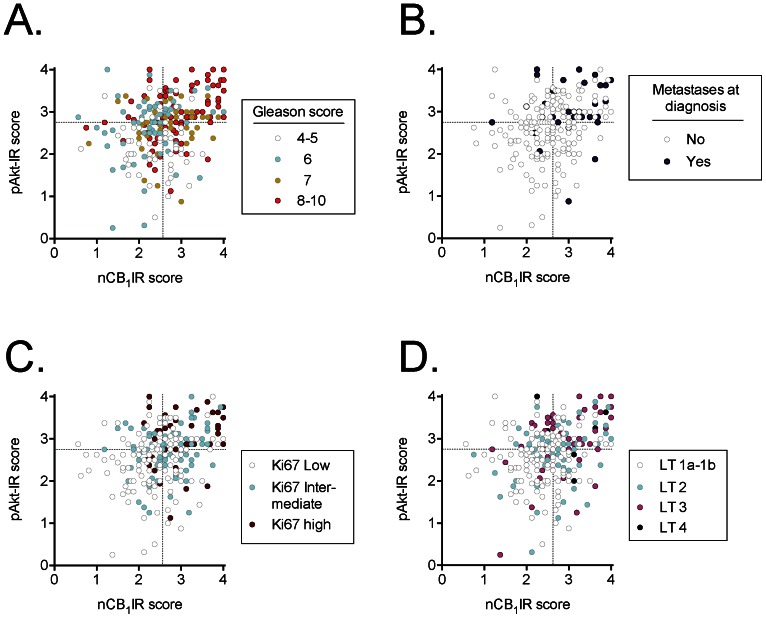
Scatter plots of cases scored for tumour nCB_1_IR (abscissae) and pAkt-IR (ordinates) and sorted on the basis of A, Gleason score (GS); B, absence or presence of metastases at diagnosis; C, Ki-67 index; D, tumour grade (LT). The Ki67 index is a continuous variable ranging from 0–48% in the dataset [34]. The tranches were chosen here for illustrative purposes but represent the bottom 50% (“Ki67 Low”), the 50–75% (“Ki67 intermediate”) and the top 25% (“Ki67 high”). The dotted lines in the figures show the median scores for nCB_1_IR and pAkt-IR for the dataset.

**Figure 3:**
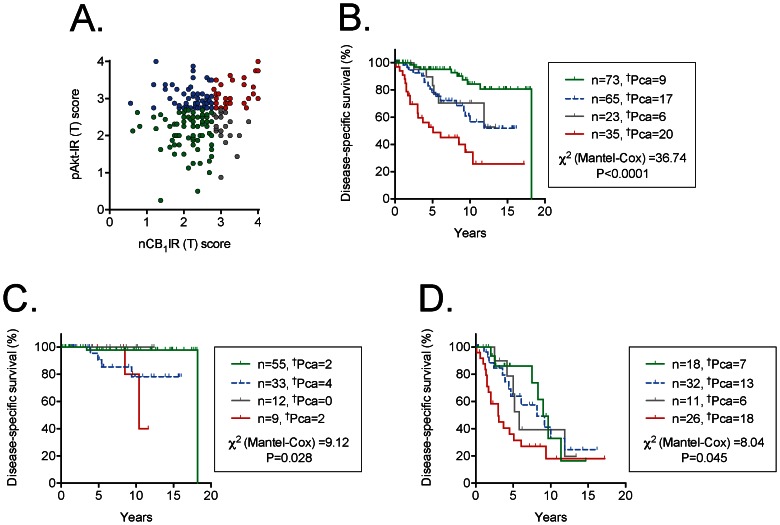
Scatter plots and Kaplan-Meier plots for the cases who were followed by expectancy and who had been scored for both nCB_1_IR and pAkt-IR. Panel A shows a scatter plot of the individual cases, so that the group names in the other Panels are easier to follow. In the Kaplan-Meier plots shown in Panels B to D, ^†^Pca refers to the number of patients who died as a result of their prostate cancer during the follow-up period. The 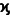 ^2^ values are for the log-rank (Mantel-Cox) tests, with the P values shown. Panel B, all cases; Panel C, Gleason score 4–6 cases; Panel D, Gleason score 7–10 cases.

We have previously reported that both tumour CB_1_IR and pAkt-IR scores in this patient material are associated with the severity of the disease at diagnosis [27], [32]. The combination of the two parameters, however, has not been studied. In Fig. 2, the individual values for cases scored for both CB_1_IR and pAkt-IR are colour-coded on the basis of their clinical/histopathological data (Gleason scores, incidence of metastases at diagnosis, tumour stage and Ki67 index [a measure of cell proliferation]). The graphs are divided into quadrants based upon the median scores for the two parameters. The scatter plots serve the additional purpose of demonstrating that although the correlation coefficient between CB_1_IR and pAkt-IR is highly significant (see above), it is a long way indeed from explaining the biological variance of the dataset. For the cases grouped as Gleason scores 4–5, 6 or 7, there was no obvious relationship between the Gleason score and the position on the graph. However, cases with Gleason scores 8–10 congregated in the top right quadrant of the graph (Fig. 3A). Similarly, cases where metastases were found at diagnosis also tended to congregate at the top right-hand part of the graph (Fig. 2B), as did cases with a higher rate of cell proliferation (Fig. 2C) and the tumour grade (Fig. 2D).

The quadrants in the figures are based on median splits of the two parameters, and contingency analyses of the data indicated that the distribution of the clinical parameters was significantly different in the quadrants (Table 2). Thus, for example, the % of the cases with Gleason scores 8–10 ranged from 19% (bottom left quadrant) to 59% (top right quadrant) and similar patterns were seen for the incidence of metastases at diagnosis, the tumour stage, the percentage of the specimens that contained tumour (%ca) and the Ki67-index (Table 2).

**Table 2:** Age, Gleason scores, incidence of metastases at diagnosis and tumour Ki67-IR at diagnosis for the cases divided on the basis of median splits of the tumour nCB_1_IR and pAkt-IR scores.

Quadrant: (see Fig 3A)		Group A (bottom left)	Group B (bottom right)	Group C (top left)	Group D (top right)	P value
*Age in years*						
median (range)		74 (56–88)	73 (52–87)	75 (62–92)	74 (51–95)	0.79^a^
{n}		{74}	{52}	{57}	{91}	0.79^a^
Number (%^b^) of cases with:						
*Gleason score*	4–5	24 (32%)	22 (42%)	6 (11%)	5 (5%)	
	6	24 (32%)	5 (10%)	24 (42%)	12 (13%)	<0.0001^c^
	7	12 (16%)	9 (17%)	10 (18%)	20 (22%)	
	8–10	14 (19%)	16 (31%)	17 (30%)	54 (59%)	
*Metastases at diagnosis*	No	55 (96%)	37 (93%)	36 (86%)	52 (68%)	<0.0001^c^
	Yes	2 (4%)	3 (8%)	6 (14%)	24 (32%)	
*Tumour stage*	1a–1b	49 (66%)	28 (54%)	26 (46%)	28 (31%)	
	2	15 (20%)	16 (31%)	15 (27%)	25 (28%)	<0.001^c^
	3	9 (12%)	6 (12%)	12 (21%)	33 (37%)	
	4	1 (1%)	2 (4%)	3 (5%)	4 (4%)	
*%ca* d						
median (25–75)		12.5 (10–62.5)	20 (10–87.5)	30 (10–77.5)	70 (30–90)	<0.0001^a^
{n}		{74}	{52}	{57}	{91}	
*Ki-67 index (%)*						
median (25–75)		1.7 (0.4–3.0)	2.7 (0.9–3.7)	3.0 (1.4–5.6)	3.9 (2.3–10)	<0.0001^c^
{n}		{74}	{51}	{56}	{89}	

“Bottom left”, “Bottom right”, “top left” and “top right” refer to the quadrants shown in Fig. 2A.

a Kruskal-Wallis test.

b The % value refers to the % of cases for the pAkt-IR/CB_1_IR group in question (i.e. vertical numbers add up to 100%).

c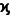 ^2^ test.

d percentage of the specimen that contained tumour. “median (25–75)” refers to the median values, with the 25–75^th^ quartiles given in brackets.

Although dramatic, the data shown in Fig. 2 and Table 2 do not indicate whether the associations between the two biochemical markers and the clinical data are simply additive or whether there is an interaction present. This was investigated for three of the parameters (Gleason score, %ca and the Ki67-index) using ordinal regression (cumulative logit model). For regressions without an interaction parameter, the contributions of pAkt-IR and nCB_1_IR were significant, as expected (Table 3). However, significant contributions of the interaction term pAkt-IR×nCB_1_IR were also seen for Gleason score and %ca when they were included in the analysis (Table 3).

**Table 3:** Ordinal regression analyses with tumour pAkt-IR and nCB_1_IR as the independent variables and the Gleason score, %ca or Ki67-index as the dependent variable.

	Main effects model		Interaction term included	
	Estimate	Z-value	Estimate	Z-value
*Gleason score*				
pAkt-IR	1.17±0.23	5.01	−0.40±0.83	−0.48
nCB_1_IR	0.49±0.21	2.36	−1.30±0.93	−1.41
pAkt-IR×nCB_1_IR			0.66±0.34	1.97
*%ca*				
pAkt-IR	0.44±0.21	2.09	−2.37±0.86	−2.74
nCB_1_IR	0.86±0.21	4.02	−2.18±0.94	−2.33
pAkt-IR×nCB_1_IR			1.16±0.35	3.28
*Ki67-index*				
pAkt-IR	0.94±0.23	4.05	−0.16±0.87	−0.19
nCB_1_IR	0.51±0.21	2.43	−0.69±0.94	−0.73
pAkt-IR×nCB_1_IR			0.45±0.35	1.31

The coefficients (± standard error) were determined from ordinal regressions (cumulative logit model) undertaken in the R statistical package (function vglm in the VGAM bundle). Gleason scores were divided as 4–5, 6, 7 and 8–10. The %ca and Ki67-index scores were divided into quartiles. In every case, the assumption of proportional odds was upheld. Significance levels for the Z-values are: ±1.96 for P = 0.05, ±2.58 for P = 0.01 and ±3.29 for P = 0.001. Note that in the analyses, the values returned for a variable in the main effects model represent its total effect when the other variable is included. In the interaction term model, the value for the individual variable is for a constant value of the other variable. The model returns three intercept coefficients for each level of the response, the reference level being the lowest level. These were as follows: Gleason score main effects model, −3.06, −4.51 and −5.52; model with interaction, 1.16, −0.30, −1.32; %ca main effects model, −1.66, −2.95, −4.01; model with interaction, 5.69, 4.36, 3.26; Ki67-index main effects model, −2.47, −3.70, −4.92; model with interaction, 0.41, −0.82, −2.04.

### Association of nCB_1_IR and pAkt-IR with disease-specific survival

Approximately two-thirds of the cases in the database had been followed by expectancy after diagnosis, this being the standard treatment protocol at the time in Sweden. These cases can provide useful information with respect to the association of a given biomarker with disease-specific survival. This has been reported previously for both pAkt-IR and the original CB_1_IR scores [27], [32]. The interaction between the two variables has, however, not been investigated. In order to do this, we have used the optimal cut-offs for the two parameters (<2.75 and ≥2.75 for pAkt-IR [32] and the Youdin cut-off value for nCB_1_IR (≤2.75 and >2.75). For all 196 patients followed by expectancy and scored for both nCB_1_IR and pAkt-IR (Fig. 3A shows the distribution of values), there was a clear pattern whereby the cases with scores of both parameters above the cut-off values had a poorer prognosis than the other three groups (Fig. 3B). A bivariate Cox proportional-hazards regression analysis indicated that the prognostic information provided by nCB_1_IR was additive to that provided by pAkt-IR (Table 4). However, this is not surprising given that the groups have different proportions of cases with different Gleason scores (see Fig. 2A). However, with Gleason scores (as a median split) were taken into consideration, the two parameters retained their additivity (Trivariate main effects analysis, Table 4), and this is reflected in the Kaplan-Meier plots of the Gleason score 7–10 cases (Fig. 3D). The interaction term pAkt-IR×nCB_1_IR, however, was not significant in either the bivariate or trivariate analyses (Table 4).

**Table 4:** COX proportional-hazards regression analyses for tumour nCB_1_IR and pAkt-IR for patients with were followed by expectancy.

	No. below/above cutoff	Exp(B)	95% CI for Exp(B)	P value
*Univariate*				
pAkt-IR	101/103	3.17	1.77–5.70	0.00011
nCB_1_IR	198/73	3.47	2.13–5.67	<0.00001
*Bivariate, main effects*				
pAkt-IR	96/100	3.20	1.72–5.95	0.00024
nCB_1_IR	138/58	2.76	1.58–4.81	0.00036
*Bivariate with interaction term*				
pAkt-IR	96/100	3.23	1.39–7.51	0.0063
nCB_1_IR	138/58	2.80	0.97–8.09	0.057
pAkt-IR×nCB_1_IR		0.98	0.28–3.39	0.97
*Trivariate, controlling for Gleason score, main effects*				
Gleason score	109/87	10.05	4.41–22.92	<0.00001
pAkt-IR	96/100	2.06	1.10–3.85	0.024
nCB_1_IR	138/58	1.88	1.07–3.28	0.027
*Trivariate, controlling for Gleason score, with interaction term*				
Gleason score	109/87	10.28	4.48–23.61	<0.00001
pAkt-IR	96/100	1.76	0.74–4.15	0.20
nCB_1_IR	138/58	1.48	0.50–4.34	0.48
pAkt-IR×nCB_1_IR		1.38	0.40–4.84	0.61

The cut-off values used were: nCB_1_IR, ≤2.75 and >2.75; pAkt-IR, <2.75 and ≥2.75 (from [32]); Gleason score 4–6 and 7–10. Exp(B) refers to the increase in risk from below to above the cut-off, i.e. the value below the cut-off is set to unity; 95% CI, 95% confidence interval.

## Discussion

In the present study we have explored the relationship between pAkt and CB_1_ in prostate cancer tumours and cell lines in order to investigate the possibility that there is an “Akt switch” in prostate cancer. At the outset, it is perhaps worth commenting on the fact that activation of CB_1_ receptors is considered here in terms of cancer cell survival, whereas many (but not all) studies in cell lines point to deleterious effects of cannabinoids upon cancer cells [1]–[5], [39]. However, the effects of cannabinoids upon tumour cells may be more complicated. This has been demonstrated elegantly in serum-starved NCI-H292 lung cancer cells: 300 nM Δ^9^-tetrahydrocannabinol (THC) produces a robust increase in cell proliferation, as assessed by a thymidine incorporation assay, whereas higher concentrations of THC (4–10 µM) produce a significant apoptosis [40]. A mitogenic effect of submicromolar concentrations of 50 and 100 nM THC upon thymidine incorporation was seen in PC-3 prostate cancer cells whereas 500 nM THC was without effect [10]. Higher concentrations of THC produce apoptosis in these cells, although this appears to be mediated by a CB receptor-independent mechanism [15]. It is notable that studies investigating the antiproliferative effects of cannabinoids often use micro- rather than nano-molar concentrations of the ligands (for discussion, see [2], [39]). Finally, antiproliferative effects of CB_1_ receptor antagonists/inverse agonists such as rimonabant have also been reported for cancer cell lines [41], [42], although it is not clear whether such effects are due to blockade of CB_1_ receptors or off-target actions of the compounds.

There are two main findings of the study, which are discussed in turn:

### CB_1_ receptor expression and pAkt expression are positively correlated in prostate cancer

One of the predictions of the pAkt switch model is that the expression of CB_1_ receptors should be positively correlated to pAkt in tumour cells. pAkt is a downstream effector molecule for a wide range of signalling pathways. This raises the risk of a false negative, where an association between CB_1_ receptors and pAkt is lost in the noise. However, a highly significant positive correlation between CB_1_IR and pAktIR was seen and the correlation was retained at the level of individual cores. Further, the correlation remained significant even when controlled for two other receptors known to couple to Akt, namely pEGFR and ErbB2 [36], [37].

### CB_1_IR and pAkt-IR associate with disease severity at diagnosis

Several studies have investigated the association of pAkt-IR with disease severity as assessed by the Gleason score, the tumour stage and the Ki67 index [32], [43]–[46]. In general, a high pAkt-IR is associated with a more severe form of the disease at diagnosis, although there are differences between the cohorts (for discussion, see [32]). With respect to CB_1_IR, two cohorts have been investigated. In our study using the present large cohort of samples obtained at diagnosis following transurethral resection for lower urinary tract symptoms, a clear association with the Gleason score and the other histopathological measures was found [27]. In contrast, in a much smaller (n = 35) cohort of samples obtained at prostatectomy, no clear association between CB_1_ receptor expression (assessed in Western blot experiments and by QT-PCR) and the Gleason grade was seen, although the expression levels were higher than seen for control tissue [28] Whether or not the patients received other treatments prior to prostatectomy was not indicated.

Given that both CB_1_IR and pAkt-IR are associated with disease severity in our cohort, it would be expected that cases with high scores of both markers would have an over-representation of Gleason scores 8–10 and the other histopathological parameters of disease severity. This was indeed found. However, with respect to the Gleason score and the percentage of the sample that was tumour associated, an interaction between nCB_1_IR and pAkt-IR was seen. Activation of Akt produces a variety of cellular effects, including inactivation of the pro-apoptotic protein BAD and an increased growth of prostate cancer cells in a xenograft model [47], [48]. Our findings are consistent with a model whereby a high CB_1_ receptor expression feed in to increase Akt signalling [30] over and above that due to other signalling pathways, thereby increasing the pathological nature of the tumour cells and hence the disease severity. Exactly how CB_1_ receptor activation feeds in to affect Akt signalling would best be investigated in cancer cell lines. However, in Chinese hamster ovary cells transfected with CB_1_ receptors, CB receptor agonists such as THC (1 µM), CP55,940 (25 nM) and HU-210 (25 nM) produce a robust activation of Akt. The effect of THC, which was not seen in the wild-type cells, was blocked by rimonabant, pertussis toxin and by wortmannin, indicative of a pathway from CB_1_ receptors involving G_i_/G_o_ receptors and phosphoinositide 3′-kinase [49]. Phosphorylation of Akt in PC-3 prostate cancer cells following incubation with THC (100 nM) or *R*-methanandamide (the hydrolysis-resistant analogue of anandamide, 100 nM) is also blocked by inhibition of phosphoinositide 3-kinase [10], suggesting that a similar pathway may be operative. Interestingly, in a variety of cell lines, THC (1 µM) and HU210 (50 nM) produce a rapid transactivation of EGFR (and in the case of THC and NCI-H292 lung cancer cells, of ErbB2) in a metalloprotease-dependent manner [40]; cannabinoid activation of Akt in the NCI-H292 cells and in SCC-9 squamous cell carcinoma cells was blocked by both EGFR- and metalloprotease inhibitors [40]. If similar mechanisms are operative in prostate tumours, they would provide an explanation for the present data.

A final note concerns the prognostic usefulness of CB_1_ receptor and pAkt expression. We have previously reported that the CB_1_IR provided prognostic information with respect to disease-specific survival that was additive to the Gleason score [27] and this was, unsurprisingly, confirmed upon rescoring the samples. Thus, the CB_1_ receptor has promise as a prognostic biomarker in prostate cancer, and it is to be hoped that independent corroboration of our finding in well-characterised patient cohorts will be forthcoming. The prognostic properties of pAkt have been demonstrated in several studies [32], [43], [50], [51] (but see [46]), but on the basis of our data its clinical utility is limited, since it provides no prognostic information for Gleason score 6–7 patients [32]. In the present study, using a simple median split of the Gleason scores, we found that nCB_1_IR and pAkt-IR provide additive prognostic information, but there was no evidence of an interaction between the two parameters with disease-specific survival as outcome measure.

In conclusion, the present study provides data that is consistent with, but does not prove, the hypothesis that at a high CB_1_ receptor expression, the Akt signalling pathway becomes operative in prostate cancer.
